# Treatment Outcomes Differ for Racial and Ethnic Minorities with Advanced-Stage Laryngeal Cancer: A Florida Cancer Data System Analysis

**DOI:** 10.1158/2767-9764.CRC-25-0239

**Published:** 2025-08-11

**Authors:** Caretia J. Washington, Chayil C. Lattimore, Jimmy J. Brown, Natalie L. Silver, Dejana Braithwaite, Kristianna M. Fredenburg, Shama D. Karanth

**Affiliations:** 1 Department of Epidemiology, University of Florida College of Public Health and Health Professions and College of Medicine, Gainesville, Florida.; 2 Department of Pathology, Immunology, and Laboratory Medicine, University of Florida, Gainesville, Florida.; 3 Department of Otolaryngology, University of Florida College of Medicine, Gainesville, Florida.; 4 Department of Otolaryngology - Head and Neck Surgery, Cleveland Clinic, Cleveland, Ohio.; 5 Center for Immunotherapy and Precision Immuno-Oncology, Cleveland Clinic, Cleveland, Ohio.; 6 University of Florida Health Cancer Center, Gainesville, Florida.; 7 Division of Population Health Sciences, Department of Surgery, College of Medicine, University of Florida, Gainesville, Florida.

## Abstract

A disparity persists in advanced-stage laryngeal cancer outcomes, in which Black patients experience lower survival rates. We examined real-world treatment patterns and outcomes by race and ethnicity in patients with advanced-stage laryngeal cancer in Florida. Data were abstracted from the Florida Cancer Data System on non-Hispanic (NH)-White, Hispanic, and NH-Black patients with advanced-stage laryngeal cancer from 2009 to 2020. Kaplan–Meier curves estimated survival by race and ethnicity. Multivariable Cox proportional hazard models calculated HRs and 95% confidence intervals (95% CI) to examine the association of race and ethnicity with treatment receipt and mortality. Cox proportional hazard models were adjusted for sociodemographic and tumor characteristics. A total of 4,316 participants with advanced-stage laryngeal cancer (75.3% NH-White, 13.1% Hispanic, and 11.6% NH-Black) were included in the analysis. In age- and sex-adjusted models, NH-Black patients had a higher risk of death (HR = 1.21; 95% CI, 1.08–1.35) compared with NH-White patients, whereas Hispanic patients had a lower risk (HR = 0.80; 95% CI, 0.71–0.90). After controlling for sociodemographic factors, mortality differences between NH-Black and NH-White patients were not statistically significant (HR = 1.12; 95% CI, 1.00–1.26). However, in treatment-stratified analyses, specifically in patients who received chemoradiation, NH-Black patients had a higher risk of death (HR = 1.25; 95% CI, 1.02–1.52) compared with NH-White patients. In conclusion, NH-Black patients with advanced-stage laryngeal cancer who underwent chemoradiation had higher mortality compared with NH-White patients, whereas Hispanic patients had lower mortality. Investigating factors such as healthcare access, comorbidities, and treatment response may help address these disparities.

**Significance:**

This study provides important insight into racial and ethnic disparities in treatment outcomes and mortality risk among patients with advanced-stage laryngeal cancer in a real-world setting. Our findings underscore the need for a comprehensive approach to understanding outcome differences, considering the interplay of healthcare access, clinical factors, and treatment quality that influence patient care and survival.

## Introduction

Laryngeal cancer comprises one thirds of all head and neck cancers with more than 13,000 cases projected in the United States for 2025 (1). The 5-year overall survival (OS) rate is around 62%; however, among patients with advanced-stage disease, the 5-year OS rate is markedly lower at 30% to 40% (2, 3). Black patients with advanced-stage laryngeal cancer have maintained the lowest OS rate for several decades (1), and because approximately 60% of patients with laryngeal cancer present with advanced-stage disease (2), understanding the basis of these persistent cancer disparities is critical.

The standard management of advanced-stage laryngeal squamous cell carcinoma, prior to 1991, was total laryngectomy. However, the focused shifted toward laryngeal preservation as the preferred definitive treatment based on the findings of the VA Larynx and RTOG 91-11 clinical trial (4, 5). Thus, for the past few decades, the standard of care for most patients with advanced-stage disease has been concurrent chemoradiation with the goal of preserving laryngeal function (6). Despite this shift, racial and ethnic disparities in treatment patterns persist. Observational studies have reported that Black patients with advanced-stage disease, particularly those with distant metastasis, are not only less likely to receive definitive treatment but are also more likely to go without treatment (7–11). Although treatment disparities are narrowing, continued differences in management have been ascribed to social factors, including lack of family support, financial barriers, and transportation (9).

The interplay of structural barriers and socioeconomic factors has been implicated in disparate survival outcomes (12, 13). Notably, Black patients that have poor OS are more likely to present with advanced-stage laryngeal cancer, be unmarried, and have lower socioeconomic status (SES), which is associated with reduced access to timely and high-quality care (7, 14, 15). These factors correlate with residing in lower-income neighborhoods and having government insurance or no insurance, further exacerbating disparities in treatment access. African Americans also experience the longest time to first treatment among patients with head and neck cancer, contributing to their higher rates of cancer-specific mortality (16).

However, the disparity in laryngeal cancer survival outcomes cannot be fully explained by structural, sociodemographic, and treatment barriers alone (12, 17, 18). Emerging evidence suggests that differences in treatment response may also play a role (17). For example, a recent study by Liu and colleagues (17) reported that Black patients participating in clinical trials—in which sociodemographic factors are controlled—were consistently more likely to experience worse survival compared with White participants in the same study arms, highlighting the need to explore additional contributors such as biological and environmental factors.

As disparities in laryngeal cancer are especially pronounced in Florida, where the mortality risk is 80% higher amongst high-poverty communities compared with low poverty communities (19), the purpose of our study was to evaluate the association of race and ethnicity, sociodemographic characteristics, and treatment strategy on survival outcomes for Floridians with advanced-stage laryngeal cancer in a real-world setting.

## Materials and Methods

### Data source

The data for this study were obtained from the Florida Cancer Data System (FCDS), which was established in 1978 by the Florida Statute as a statewide central cancer registry. The FCDS is supported by the National Program of Cancer Registries and administered by the Centers for Disease Control and Prevention. The data include patient demographics, tumor characteristics, treatment receipt, and survival data. This study was considered exempt by the University of Florida Institutional Review Board as the data obtained were deidentified.

### Study design

This is a retrospective cohort study design, and due to the nature of its observational design, randomization and blinding were not necessary. Additionally, power analysis is not required for this study because the focus is on analyzing existing data rather than estimating the effects of an intervention.

### Study cohort

The study cohort included patients who met the following criteria: (i) had a diagnosis of laryngeal cancer (International Classification of Diseases for Oncology, Third Edition, topography codes: C320, C321, C322, C323, C328, and C329); (ii) were staged as either regionalized or distant (advanced stage) according to the Surveillance, Epidemiology, and End Results (SEER) Summary Staging 2000 and SEER Summary Staging 2018 criteria; (iii) were diagnosed between 2009 and 2020; (iv) had squamous cell carcinoma as the histologic type; and (v) had no missing data for race and ethnicity, treatment status, or survival time. Regionalized stage was defined as one of the following: (i) regional by direct extension only (tumor extension beyond the site of origin without lymph node involvement), (ii) regional lymph node(s) only, or (iii) regional by both direct extension and regional lymph node(s) involvement. Distant stage was defined as metastatic disease characterized by tumor cells spreading beyond regional lymph nodes or adjacent tissues to distant parts of the body. Patients with distant metastasis were included in the analysis to comprehensively evaluate the impact of advanced-stage disease (stage IV) on survival outcomes.

### Exposure, outcome, and covariates

The main exposure considered in this data analysis was self-reported race and ethnicity, which was categorized into the following groups: non-Hispanic (NH)-White, Hispanic, and NH-Black. Hereafter, we exclude the NH prefix when referencing racial groups. Our outcome of interest was overall mortality, as survival time was assessed (in months) starting from the date of diagnosis until death or the last contact, whichever occurred first. Sociodemographic covariates included in this study from the FCDS were age [(<65 years and ≥65 years based on the age for Medicare eligibility (continuous age used in Cox proportional models)], biological sex (male and female), marital status (married, unmarried, and unknown), and primary payer at diagnosis [private, government, not insured, and not otherwise specified (NOS)/unknown]. Rurality was measured at the county level from the 2013 Rural–Urban Commuting Area Codes (metropolitan and nonmetropolitan). Median household income was determined by the 2008 to 2012 Florida Health Charts and categorized into above and below $48,000 (20). Educational level was measured by the percentage of the county with no high school diploma, determined by the 2010 US Census Bureau Statistical Atlas, and categorized into tertiles (13%, 7%–12.9%, and <7%). Treatment was considered part of the first course of treatment based on the North American Association of Central Cancer Registries, which involved the following variables: surgery (yes vs. no vs. NOS/unknown), radiation (yes vs. no vs. NOS/unknown), and chemotherapy (yes vs. no vs. NOS/unknown).

Treatment strategy receipt was determined based on the combinations of the treatment variables, including standard-of-care treatments (chemoradiation, surgery combined with chemoradiation, surgery and radiation, and radiation alone) and non–standard-of-care treatments (chemotherapy alone, surgery alone, surgery and chemotherapy, and no treatment). Additional covariates included primary subsite (glottis, supraglottis, subglottis, and other/NOS), tumor grade (well differentiated/moderately differentiated, poorly differentiated/undifferentiated, and unknown), and stage (regional and distant). Covariates were chosen based on *a priori* knowledge of their associations with the specified exposure and outcome.

### Statistical analysis

Differences in proportions of each potential covariate were compared by race and ethnicity using Pearson *χ*^2^ tests for categorical variables. Univariate and multivariate associations between race and ethnicity and survival were examined using Kaplan–Meier plots and Cox proportional hazards models. Kaplan–Meier curves were used to estimate survival probability by race and ethnicity. Differences in survival probabilities were assessed through the log-rank test. Reasons for the absence of surgery were also stratified based on race and ethnicity. The number of deaths and mortality rates (expressed as deaths per 10 person-years) were summarized for the entire study population, categorized by race and ethnicity and further stratified by treatment receipt. In addition, OS percentages were calculated at 1-, 3-, and 5-year follow-up by race and ethnicity and treatment strategy. Cox proportional hazard models with survival time as the time scale were used to estimate the risk of mortality associated with race and ethnicity (with White as the reference group), adjusting for the aforementioned covariates. Risk was expressed as hazard ratios (HRs) and 95% confidence intervals (95% CI). Additionally, descriptive statistics were conducted for the subpopulation of patients who did not receive any treatment. This subpopulation served as the analytic cohort for the race- and ethnicity-stratified Cox proportional hazards model. The level of significance was set at 0.05, and all reported *P* values are two-sided.

After computing continuous age- and sex-adjusted Cox proportional hazard models for race and ethnicity, sociodemographic covariates were added to the model. The model was then further adjusted for primary site and stage. For models not stratified by treatment strategy, treatment was additionally adjusted. These were known prognostic variables and variables that showed statistically significant relations with either the independent or dependent variable (with a *P* < 0.01 level of significance). Although grade is presented in the patient characteristics table, it was not identified as a statistically significant confounder in our multivariable models and was therefore excluded from the final analyses. Additionally, descriptive statistics for the complete case population, which excluded patients with missing information for marital status and primary payer at diagnosis, were conducted. This cohort was used for the complete case analysis. All Cox proportional hazard models were tested for proportionality of hazards using Schoenfeld residuals. When this assumption was violated, stratified proportional hazard models were fitted; no material differences in HRs were observed. All statistical analyses were conducted using SAS 9.4.

### Data availability

The data underlying this article were provided by the FCDS by permission. Data will be shared on request to the corresponding author with permission of the FCDS.

## Results

**Figure 1:**
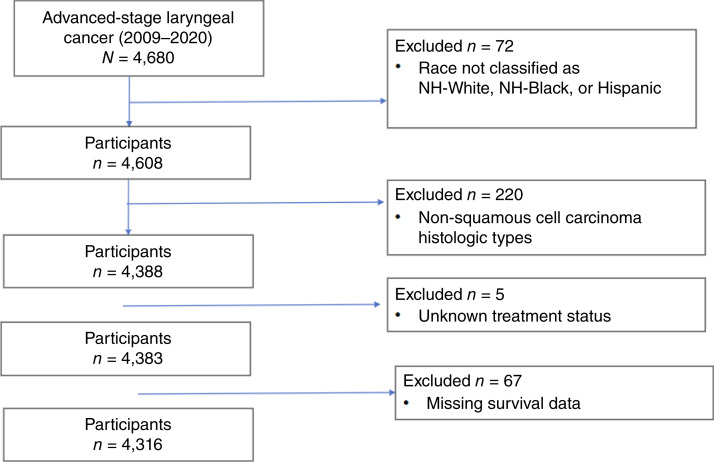
Flow chart of study participants.

The final study cohort included 4,316 patients diagnosed with advanced-stage laryngeal cancer from 2009 to 2020 (75.3% White, 13.1% Hispanic, and 11.6% Black; Fig. 1). Table 1 shows that a majority of patients were less than 65 years old at time of diagnosis (58.5%), male (79.7%), had supraglottic tumors (53.1%), and presented with regional stage disease (65.3%). Additionally, a majority of the cohort were more likely to be unmarried (54.7%), have government insurance (64.5%), live in a metropolitan area (94.1%), and have an income less than $48,000 per annum (57.9%). However, compared with the reference population (White patients), Black patients were more likely to be less than 65 years old at the time of diagnosis (65.7% vs. 58.0%), more likely to be unmarried (70.1% vs. 52.5%), and less likely to have private insurance (16.8% vs. 22.4%). Interestingly, Black patients were also more likely than White patients to live in a county with a median household income greater than $48,000 (45.7% vs. 43.5%). Conversely, Hispanic patients were more like to live in a county with a median household income of less than $48,000 (69.4%) and with 13% or more of the population lacking a high school diploma (67.0%). Notably, Black (39.5%) and Hispanic (39.1%) patients were also more likely than White patients (33.3%) to present with distant-stage disease.

**Table 1:** Sociodemographic and cancer characteristics of patients with regional and distant-staged laryngeal cancer

​	Overall (%)	Race *n* (%)		
	*N* = 4,316	NH-White*N* = 3,251 (75.3)	Hispanic*N* = 566 (13.1)	NH-Black*N* = 499 (11.6)
Age	​	​	​	​
<65 years	2,523 (58.5)	1,887 (58.0)	308 (54.4)	328 (65.7)
65+ years	1,793 (41.54)	1,364 (42.0)	258 (45.6)	171 (34.3)
Sex	​	​	​	​
Male	3,439 (79.7)	2,534 (78.0)	485 (85.7)	420 (84.2)
Female	877 (20.3)	717 (22.1)	81 (14.3)	79 (15.8)
Marital status	​	​	​	​
Married	1,786 (41.4)	1,424 (43.8)	238 (42.1)	124 (24.9)
Unmarried	2,361 (54.7)	1,705 (52.5)	306 (54.1)	350 (70.1)
Unknown	169 (3.9)	122 (3.8)	22 (3.9)	25 (5.0)
Primary insurance	​	​	​	​
Private	939 (21.8)	729 (22.4)	126 (22.3)	84 (16.8)
Government	2,783 (64.5)	2,107 (64.8)	344 (60.8)	332 (66.5)
Not insured	345 (8.0)	233 (7.2)	61 (10.8)	51 (10.2)
NOS/unknown	249 (5.8)	182 (5.6)	35 (6.2)	32 (6.4)
Rurality	​	​	​	​
Metropolitan	4,060 (94.1)	3,042 (93.6)	556 (98.2)	462 (92.6)
Nonmetropolitan	256 (5.9)	209 (6.4)	10 (1.8)	37 (7.4)
County median household income	​	​	​	​
<$48,000	2,500 (57.9)	1,836 (56.5)	393 (69.4)	271 (54.3)
≥48,000	1,816 (42.1)	1,415 (43.5)	173 (30.6)	228 (45.7)
Percentage of people in the county without high school diploma	​	​	​	​
13% or more	1,366 (31.7)	837 (25.8)	379 (67.0)	150 (30.1)
7%–12.9%	2,830 (65.6)	2,308 (71.0)	180 (31.8)	342 (68.5)
<7%	120 (2.8)	106 (3.3)	7 (1.2)	7 (1.4)
Primary subsite	​	​	​	​
Glottis	1,155 (26.8)	833 (25.6)	198 (35.0)	124 (24.9)
Supraglottis	2,290 (53.1)	1,774 (54.6)	251 (44.4)	265 (53.1)
Subglottis	88 (2.0)	70 (2.2)	10 (1.8)	8 (1.6)
Other/NOS	783 (18.1)	574 (17.7)	107 (18.9)	102 (20.4)
Grade	​	​	​	​
Well differentiated/moderately differentiated	1,694 (39.3)	1,253 (38.5)	232 (41.0)	209 (41.9)
Poorly differentiated/undifferentiated	785 (18.2)	596 (18.3)	105 (18.6)	84 (16.8)
Unknown	1,837 (42.6)	1,402 (43.1)	229 (40.5)	206 (41.3)
Stage	​	​	​	​
Regional	2,816 (65.3)	2,169 (66.7)	345 (61.0)	302 (60.5)
Distant	1,500 (34.8)	1,082 (33.3)	221 (39.1)	197 (39.5)

Table 2 displays treatment differences by race and ethnicity. For all racial and ethnic groups, chemoradiation was the most common treatment modality used among standard treatments (61.4%). When considering treatment differences by race and ethnicity, Hispanic (50.7%) and NH-Black (49.3%) patients were more likely to receive nonstandard treatment as compared with NH-White patients (52.9%). Hispanic patients were also the most likely to receive surgery alone among nonstandard treatments (29.3%) and were less likely to refuse surgery (0.6%; Table 3). Most commonly, patients in Florida treated for advanced-stage laryngeal cancer were less likely to receive surgery because it was not part of the treatment plan (96.9%; Table 3).

**Table 2:** Treatment strategies for patients with advanced-stage laryngeal cancer by race and ethnicity

Treatment strategy	Overall (%)	Race *n* (%)		
	*N* = 4,316	NH-White*N* = 3,251 (75.3)	Hispanic*N* = 566 (13.1)	NH-Black*N* = 499 (11.6)
Standard treatment	2,250 (52.1)	1,718 (52.9)	279 (49.3)	253 (50.7)
Chemoradiation	1,382 (61.4)	1,081 (62.9)	158 (56.6)	143 (56.5)
Surgery and chemoradiation	257 (15.0)	57 (20.4)	46 (18.2)	257 (15.0)
Surgery and radiation	245 (10.9)	171 (10.0)	38 (13.6)	36 (14.2)
Radiation alone	263 (11.7)	209 (12.2)	26 (9.3)	28 (11.1)
Nonstandard treatment	2,066 (47.9)	1,533 (47.2)	287 (50.7)	246 (49.3)
Chemotherapy alone	698 (33.8)	536 (35.0)	87 (30.3)	75 (30.5)
Surgery alone	519 (25.1)	378 (24.7)	84 (29.3)	57 (23.2)
Surgery and chemotherapy	157 (7.6)	102 (6.7)	28 (9.8)	27 (11.0)
No treatment	692 (33.5)	517 (33.7)	88 (30.7)	87 (35.4)

**Table 3:** Reasons for no surgery among patients with advanced-stage laryngeal cancer

​	Overall (%)	Race *n* (%)		
	*N* = 3,034	NH-White*N* = 2,342 (77.2)	Hispanic*N* = 359 (11.8)	NH-Black*N* = 333 (11.0)
Reason for no surgery				
Surgery not performed because not part of first treatment planned	2,941 (96.9)	2,269 (96.9)	352 (98.1)	320 (96.1)
Surgery refused by patient	31 (1.02)	26 (1.1)	2 (0.6)	3 (0.9)
Surgery not performed because contraindicated by patient risk factors	37 (1.22)	29 (1.2)	3 (0.8)	5 (1.5)
Other	25 (0.82)	18 (0.8)	2 (0.6)	5 (1.5)

Reasons for no surgery categorized as “other” include surgery not performed because patient died prior to surgery, surgery was recommended but not as part of the first-course therapy, surgery was recommended but unknown whether performed, and unknown whether surgery was recommended or performed.

**Figure 2:**
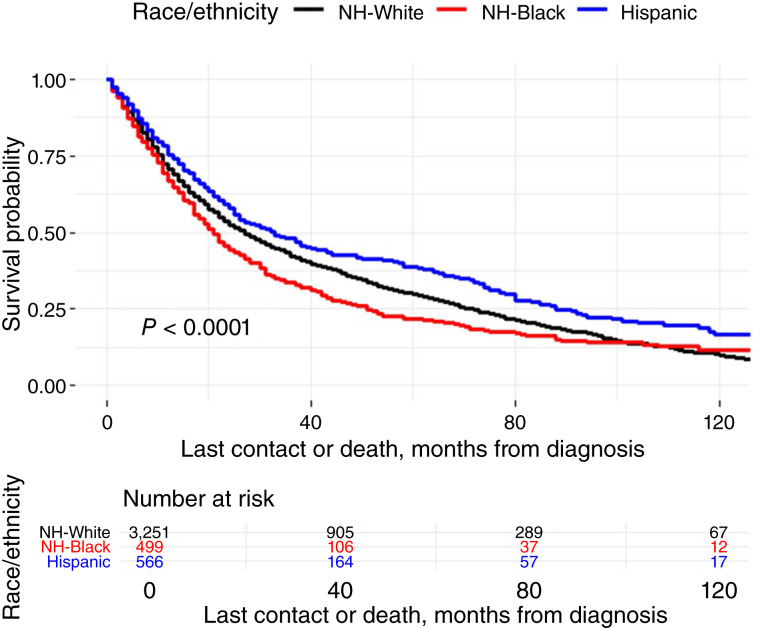
Kaplan–Meier curve of patients with advanced-stage laryngeal cancer by race and ethnicity.

In Fig. 2, among different racial and ethnic groups, Kaplan–Meier curves revealed the lowest survival probabilities in Black patients and the highest survival probabilities in Hispanic patients, with a statistically significant log-rank *P* value < 0.0001. The 1-, 3-, and 5-year OS rates were the highest among Hispanic patients and lowest among Black patients (Supplementary Table S1). At 5 years, survival was 38.7% for Hispanic, 29.7% for White, and 21.7% for Black patients. Across all groups, survival was the highest for patients treated with surgery and radiation (45.2%) and lowest for those receiving radiation alone (20.8%) or nonstandard treatment (26.6%).

Table 4 displays that the overall death rate for Black patients with advanced-stage laryngeal cancer was 3.15 deaths/10 person-years and was the lowest for Hispanic patients at 2.13 deaths/10 person-years. When adjusting for age and sex (model 1), Black patients had a higher risk of death compared with White patients, [adjusted HR (aHR) = 1.21; 95% CI, 1.08–1.35]; however, the overall risk of death for Black patients was no longer statistically significant with sociodemographic adjustments in model 2 (aHR = 1.12; 95% CI, 1.00–1.26) and tumor characteristic adjustments in model 3 (aHR = 1.08; 95% CI, 0.96–1.21). Conversely, Hispanic patients had a lower risk of death in all models compared with White patients (full model aHR = 0.77; 95% CI, 0.68–0.87).

**Table 4:** Cox proportional hazards models predicting risk of all-cause death for patients with advanced-stage laryngeal cancer, stratified by race and ethnicity and treatment strategy

Race and ethnicity	Crude death rate	Person-years	Death rate (per 10 person-years)	Model 1	Model 2	Model 3
Overall						
NH-White	2,205/3,251	8,351	2.64 (2.53–2.75)	REF	REF	REF
NH-Black	358/499	1,138	3.15 (2.83–3.49)	**1.21 (1.08–1.35)**	1.12 (1.00–1.26)	1.08 (0.96–1.21)
Hispanic	328/566	1,539	2.13 (1.91–2.37)	**0.80 (0.71–0.90)**	**0.77 (0.68–0.87)**	**0.77 (0.68–0.87)**
Chemoradiation						
NH-White	776/1,081	3,582	2.17 (2.02–2.32)	REF	REF	REF
NH-Black	113/143	414	2.73 (2.26–3.27)	**1.34 (1.10–1.64)**	**1.24 (1.02–1.52)**	**1.25 (1.02–1.53)**
Hispanic	98/158	543	1.81 (1.47–2.19)	0.832 (0.67–1.03)	**0.80 (0.64–0.99)**	**0.77 (0.62–0.96)**
Surgery and chemoradiation						
NH-White	186/257	942	1.98 (1.71–2.27)	REF	REF	REF
NH-Black	37/46	129	2.87 (2.05–3.91)	1.35 (0.94–1.93)	1.35 (0.93–1.95)	1.33 (0.92–1.93)
Hispanic	32/57	212	1.51 (1.05–2.11)	0.75 (0.51–1.09)	0.70 (0.47–1.04)	0.66 (0.44–1.00)
Surgery and radiation						
NH-White	106/171	570	1.86 (1.53–2.24)	REF	REF	REF
NH-Black	22/36	118	1.86 (1.20–2.78)	1.06 (0.67–1.68)	0.87 (0.52–1.45)	0.87 (0.52–1.46)
Hispanic	20/38	174	1.15 (0.72–1.74)	**0.56 (0.34–0.90)**	0.61 (0.36–1.04)	0.61 (0.36–1.03)
Radiation alone						
NH-White	176/209	516	3.41 (2.93–3.94)	REF	REF	REF
NH-Black	23/28	50	4.60 (2.99–6.79)	1.30 (0.84–2.01)	1.21 (0.76–1.91)	1.05 (0.66–1.69)
Hispanic	18/26	75	2.40 (1.47–3.72)	0.69 (0.42–1.19)	**0.54 (0.31–0.92)**	**0.52 (0.30–0.92)**
Nonstandard treatment						​
NH-White	961/1,533	2,742	3.51 (2.29–3.73)	REF	REF	REF
NH-Black	163/246	427	3.82 (3.26–4.44)	1.11 (0.94–1.32)	1.02 (0.86–1.21)	1.00 (0.84–1.18)
Hispanic	160/287	534	3.00 (2.56–3.49)	0.84 (0.71–1.00)	**0.83 (0.69–0.99)**	0.85 (0.71–1.02)

Bolded values indicate *P* < 0.05.

Model 1 was adjusted for continuous age and sex.

Model 2 was additionally adjusted for marital status, rurality, income, education, and insurance.

Model 3 was additionally adjusted for primary subsite and stage. Overall model was adjusted for treatment.

Crude death rate was calculated by the number of deaths divided by the total number of individuals.

Death rate was calculated as the number of deaths divided by person-years (reported as cases/10 person-years).

Nonstandard treatment includes chemotherapy alone, surgery alone, surgery followed by chemotherapy, and no treatment.

When stratified by treatment strategy, we observed that there was no difference in rate of death for Black patients when compared with White patients, with the exception of chemoradiation receipt. Black patients treated with chemoradiation experienced a higher risk of death, across all models, when compared with White patients (full model; aHR = 1.25; 95% CI, 1.02–1.53). On the other hand, Hispanic patients experienced a lower risk of death for chemoradiation receipt (aHR = 0.77; 95% CI, 0.62–0.96) as compared with White patients. Additionally, among patients who received radiation alone, Hispanic patients experienced a lower risk of death compared with White patients (full model aHR = 0.52; 95% CI, 0.30–0.92).

There were not significant differences in the risk of death in the complete case analysis, except that among patients receiving chemoradiation, Hispanic patients no longer had a significantly lower risk of mortality. Overall, patients who did not receive treatment had characteristics similar to the full cohort, although a higher proportion were younger (<65 years: 54.8%) and more had government insurance (70.5%; Supplementary Table S2). Among individuals who did not receive any treatment, Hispanic patients had a lower risk of death compared with White patients (full model; aHR = 0.67; 95% CI, 0.50–0.90; Supplementary Table S3).

Descriptive statistics for the complete case analysis (Supplementary Table S4) showed no major differences from the overall population. In the complete case analysis, no significant differences in the risk of death were observed, except among patients receiving chemoradiation. Hispanic patients no longer had a significantly higher risk of death compared with White individuals (full model, aHR = 0.82; 95% CI, 0.65–1.03; Supplementary Table S5). However, Black patients receiving chemoradiation continued to have a lower risk of death compared with their White counterparts (full model, aHR = 1.24; 95% CI, 1.01–1.54).

## Discussion

Disparities in laryngeal cancer persist, with Black patients experiencing the lowest OS (7). In our analysis of advanced-stage laryngeal cancer in Florida, Black patients initially had higher mortality risk, but this difference was no longer significant after adjusting for socioeconomic factors. However, among patients receiving chemoradiation, Black patients remained at significantly higher risk of mortality even after controlling for SES and tumor characteristics, facing a 25% increased risk compared with their White counterparts. In contrast, Hispanic patients exhibited lower overall mortality risk, a survival advantage that persisted both before treatment stratification and among those receiving chemoradiation. Additionally, among patients treated with radiation alone, Hispanic individuals experienced even better survival outcomes. These findings underscore the urgent need to investigate the multilevel mechanisms driving these divergent survival patterns, including neighborhood-level factors, comorbidities, and tumor biology.

Chemoradiation is one of the most common treatment modalities for advanced-stage laryngeal cancer (21), which is consistent with our study’s observation that most patients with advanced-stage laryngeal cancer in Florida, irrespective of race and ethnicity and insurance status, received chemoradiation. However, we also observed that Black and Hispanic patients were more likely to receive nonstandard treatments and less likely to undergo chemoradiation compared with their White counterparts. Among those treated with chemoradiation, Black patients experienced significantly higher mortality, despite adjustments for sociodemographic factors and tumor characteristics. Our observation is comparable with Liu and colleagues’ study in which they used Radiation Therapy Oncology Group clinical trial data to assess outcomes from Black participants with head and neck cancer with study arm–specific White control participants (17). The group determined that Black patients treated for head and neck cancer had significantly worse OS secondary to an increase in locoregional failure. The authors’ study provided a unique opportunity to evaluate cancer health disparities in a controlled setting. However, studies such as these do not directly translate to the real-world setting, as patients involved in clinical trials tend to receive more standardized treatment and follow-up care than the average patient (22). Moreover, real-world studies capture the complexities of routine clinical practice (23, 24), which can enhance understanding of treatment effectiveness in underrepresented populations (25).

Our study, like many others using cancer registries (26–28), provides insights into disparities in advanced-stage laryngeal cancer in a real-world setting by incorporating patient data from a state cancer registry. This is further reinforced by Molina and colleagues (26), who also used the FCDS and identified significant survival disparities among African American patients with head and neck cancer, independent of demographics, SES, comorbidities, clinical characteristics, and undertreatment. Although our study also used the FCDS, our study adds to this literature by stratifying outcomes by treatment modality, specifically within advanced-stage laryngeal cancer, using more recent data from 2009 to 2020. This approach provides deeper insight into the impact of different treatment strategies on survival disparities, offering a more up-to-date understanding of how treatment influences outcomes among various racial and ethnic groups in a real-world setting. Our findings suggest that there may be factors outside of those controlled for that influence Black patients’ response to chemoradiation. Potential factors may include neighborhood SES and enclave status, which have been found to affect group-level outcomes by providing additional context for disparities in treatment access and response (12). On a more individual context, factors such as host immune response, tumor biology, and genetic ancestry may further explain variations in survival and treatment effectiveness among different racial and ethnic groups. These considerations highlight the complex, multifactorial nature of health disparities in cancer care and underscore the need for personalized approaches to improve outcomes (29–31). Notably, Hispanic individuals with head and neck cancer have better survival across racial and ethnic populations despite having lower SES and decreased follow-up to care (32). This observation has been seen in other health conditions in a manner known as the “Hispanic paradox” (33–35). Similarly, our report indicates that Hispanic patients with advanced-stage laryngeal cancer have lower educational attainment, lower income, and an increase in distant metastatic disease. Paradoxically, Hispanic patients had lower overall mortality across all models when compared with White patients. Furthermore, Hispanic patients experienced lower mortality when treated with radiation alone. Future research is necessary to understand the basis of this survival advantage which may also be related to host factors, tumor biology, and/or genetic ancestry.

Strengths of this study include utilizing the FCDS, which provides substantial statistical power for examining racial and ethnic differences because of its large sample size and encompassing data from 2009 to 2020. In our models, we were able to adjust for many potential factors, such as sociodemographic and tumor characteristics, while taking into account several different treatment strategies that could affect the disparate outcomes for advanced-stage laryngeal cancer. Nonetheless, our study was not without limitations. We recognize the limitation of using self-reported race as a variable, which may not fully consider the underpinning of genetic ancestry. Due to the nature of the database, we were unable to control for body mass index or comorbidities. County-level information had to be extracted for area-level SES (using income level, educational level, and rurality), which may not accurately capture the specific socioeconomic or geographic characteristics of every patient, leading to potential misclassification. Additionally, whereas the SEER Summary Staging criteria allow for consistent grouping of patients across stages, they lack the granular detail of tumor–node–metastasis staging, which limits the ability to capture finer distinctions in tumor size and nodal characteristics. Also, the database only provides data on the first course of treatment, which precludes an evaluation of temporal trends in treatment modalities over the 11-year study period. Furthermore, the data are limited to the Florida Cancer Registry rather than other cancer registries or the SEER registries, which may affect the generalizability of our findings to broader populations. Finally, by assessing overall mortality instead of cancer-specific mortality, we did not account for competing risks. Despite these limitations, the comprehensive nature of the FCDS and the robust adjustments made in our models provide a strong foundation for understanding disparities in advanced-stage laryngeal cancer outcomes, offering valuable insights for future research and intervention efforts.

### Conclusions

This comprehensive cancer registry study examined the racial and ethnic differences in treatment and survival among patients with advanced-stage laryngeal cancer in a real-world setting. Our findings revealed that Black patients experience lower survival rates compared with their White counterparts in models adjusted for age and sex. Whereas adjusting for sociodemographic factors mitigated this racial disparity, the disparity persisted among patients undergoing chemoradiation, with Black patients consistently exhibiting poorer survival outcomes across all statistical models. This study underscores the importance of the development of comprehensive interventions targeting social determinants of health and clinical factors to ensure equitable access to high-quality cancer care and reduce existing disparities in treatment outcomes. Future research must also explore biological and clinical differences in advanced-stage laryngeal cancer by race and ethnicity, as there is an urgent need to advance precision medicine to enhance treatment responses and outcomes for all patients equitably.

## Supplementary Material

Supplementary Table S1 One-, Three-, and Five-year overall survival rates (%) by race and ethnicity and treatment strategy.

Supplementary Table S2 Sociodemographic and cancer characteristics for regional and distant staged laryngeal cancer patients who did not receive treatment.

Supplementary Table S3 Cox proportional hazards models predicting risk of all-cause death for patients with advanced-stage laryngeal cancer who did not receive treatment, stratified by race and ethnicity and treatment strategy.

Supplementary Table S4 Sociodemographic and cancer characteristics for regional and distant staged laryngeal cancer patients who did not receive treatment.

Supplementary Table S5 Complete Case Analysis- Cox proportional hazards models predicting risk of all-cause death for patients with advanced-stage laryngeal cancer, stratified by race and ethnicity and treatment strategy
