# Supplementary material for: Treatment Outcomes Differ for Racial and Ethnic Minorities with Advanced-Stage Laryngeal Cancer: A Florida Cancer Data System Analysis
Source: Cancer Res Commun. 2025 Aug 11;5(8):1310–8. doi: 10.1158/2767-9764.CRC-25-0239 (PMC12336365; doi:10.1158/2767-9764.CRC-25-0239)
Supplement: Supplementary Table S1 — One-, Three-, and Five-year overall survival rates (%) by race and ethnicity and treatment strategy. [file crc-25-0239_supplementary_table_s1_suppst1.docx]

**Supplementary Table S1: One-, Three-, and Five-year overall survival rates (%) by race and ethnicity and treatment strategy**

| **Race and Ethnicity** | **Overall** | **NH-White** | **NH-Black** | **Hispanic** |
| --- | --- | --- | --- | --- |
| **One-year relative survival rates (%, SE)** | | | | |
| Overall | 70.7 (0.7) | 70.5 (0.8) | 66.9 (2.2) | 75.4 (1.9) |
| Chemoradiation | 79.5 (1.1) | 79.6 (1.3) | 75.1 (24.9) | 83.1 (3.0) |
| Surgery and Chemoradiation | 82.3 (2.1) | 81.2 (2.5) | 82.1 (5.7) | 87.3 (4.5) |
| Surgery and radiation | 88.3 (2.2) | 90.3 (2.4) | 82.0 (6.7) | 85.6 (6.0) |
| Radiation alone | 63.7 (3.0) | 63.8 (3.4) | 56.5 (9.5) | 71.5 (9.1) |
| Non-standard treatment | 61.4 (1.1) | 60.8 (1.3) | 58.2 (3.3) | 67.6 (2.9) |
| **Three-year relative survival rates (%, SE)** | | | | |
| Overall | 42.0 (0.8) | 42.3 (0.9) | 33.9 (2.3) | 48.0 (2.3) |
| Chemoradiation | 48.6 (51.4) | 49.4 (1.6) | 36.3 (4.2) | 54.5 (4.2) |
| Surgery and Chemoradiation | 51.4 (2.8) | 52.3 (3.2) | 43.7 (7.7) | 53.1 (7.1) |
| Surgery and radiation | 59.7 (3.5) | 55.2 (4.2) | 59.0 (8.8) | 82.4 (6.6) |
| Radiation alone | 31.3 (3.0) | 31.3 (3.3) | 17.6 (7.9) | 45.6 (10.3) |
| Non-standard treatment | 35.0 (1.2) | 35.5 (1.4) | 28.5 (3.3) | 37.7 (3.5) |
| **Five-year relative survival rates (%, SE)** | | | | |
| Overall | 29.9 (0.8) | 29.7 (0.9) | 21.7 (2.2) | 38.7 (2.4) |
| Chemoradiation | 33.8 (1.4) | 34.2 (1.6) | 20.8 (3.7) | 43.6 (4.3) |
| Surgery and Chemoradiation | 34.4 (2.7) | 35.1 (3.2) | 25.7 (6.9) | 38.6 (7.2) |
| Surgery and radiation | 45.2 (3.6) | 41.7 (4.3) | 31.5 (8.9) | 74.9 (7.8) |
| Radiation alone | 20.8 (2.6) | 20.1 (2.9) | 8.80 (5.9) | 39.9 (10.5) |
| Non-standard treatment | 26.6 (1.3) | 26.3 (1.5) | 24.1 (3.4) | 30.2 (3.6) |

Abbreviations: SE, standard error.

Non-standard treatment includes chemotherapy alone, surgery alone, surgery followed by chemotherapy, and no treatment.
