# Supplementary material for: Treatment Outcomes Differ for Racial and Ethnic Minorities with Advanced-Stage Laryngeal Cancer: A Florida Cancer Data System Analysis
Source: Cancer Res Commun. 2025 Aug 11;5(8):1310–8. doi: 10.1158/2767-9764.CRC-25-0239 (PMC12336365; doi:10.1158/2767-9764.CRC-25-0239)
Supplement: Supplementary Table S2 — Sociodemographic and cancer characteristics for regional and distant staged laryngeal cancer patients who did not receive treatment. [file crc-25-0239_supplementary_table_s2_suppst2.docx]

**Supplementary Table S2: Sociodemographic and cancer characteristics for regional and distant staged laryngeal cancer patients who did not receive treatment.**

|  | **Overall (%)** |  | **Race n (%)** |  |  |
| --- | --- | --- | --- | --- | --- |
|  | **N= 692** | **NH-White**  **N= 517 (74.71)** | **Hispanic**  **N= 88 (12.72)** | **NH-Black**  **N=87 (12.57)** |  |
| **Age** |  |  |  |  |  |
| <65 | 313 (45.2) | 241 (46.6) | 31 (35.2) | 41 (47.1) |  |
| 65+ | 379 (54.8) | 276 (53.4) | 57 (64.8) | 46 (52.9) |  |
| **Sex** |  |  |  |  |  |
| Male | 553 (79.9) | 395 (76.4) | 77 (87.5) | 81 (93.1) |  |
| Female | 139 (20.1) | 122 (23.6) | 11 (12.5) | 6 (6.9) |  |
| **Marital Status** | | | | |  |
| Married | 242 (35.0) | 184 (35.6) | 32 (36.4) | 26 (29.9) |  |
| Unmarried | 418 (60.4) | 310 (60.0) | 50 (56.8) | 58 (66.7) |  |
| Unknown | 32 (4.6) | 23 (4.5) | 6 (6.8) | 3 (3.5) |  |
| **Primary insurance** | | | | |  |
| Private | 106 (15.3) | 81 (15.7) | 17 (19.3) | 8 (9.2) |  |
| Government | 488 (70.5) | 370 (71.6) | 54 (61.4) | 64 (73.6) |  |
| Not insured | 57 (8.2) | 38 (7.4) | 9 (10.2) | 10 (11.5) |  |
| NOS/Unknown | 41 (5.9) | 28 (5.4) | 8 (9.1) | 5 (5.8) |  |
| **Rurality** |  |  |  |  |  |
| Metropolitan | 653 (94.4) | 486 (94.0) | 86 (97.7) | 81 (93.1) |  |
| Nonmetropolitan | 39 (5.6) | 31 (6.0) | 2 (2.3) | 6 (6.9) |  |
| **County Median Household Income** | | | | |  |
| <$48,000 | 409 (59.1) | 298 (57.6) | 67 (76.1) | 44 (50.6) |  |
| ≥48,000 | 283 (40.9) | 219 (42.4) | 21 (23.9) | 43 (49.4) |  |
| **Percentage of People in the County without High School Diploma** | | | | |  |
| 13% or more | 211 (30.5) | 124 (24.0) | 64 (72.7) | 23 (26.4) |  |
| 7-12.9% | 460 (66.5) | 373 (72.2) | 23 (26.1) | 64 (73.6) |  |
| <7% | 21 (3.0) | 20 (3.9) | 1 (1.1) | 0 (0.0) |  |
| **Primary Subsite** |  |  |  |  |  |
| Glottis | 182 (26.3) | 132 (25.5) | 29 (33.0) | 21 (24.1) |  |
| Supraglottis | 352 (50.9) | 265 (51.3) | 38 (43.2) | 49 (56.3) |  |
| Subglottis | 17 (2.5) | 15 (2.9) | 0 (0.0) | 2 (2.3) |  |
| Other/NOS | 141 (20.4) | 105 (20.3) | 21 (23.9) | 15 (17.2) |  |
| **Grade** |  |  |  |  |  |
| Well differentiated/Moderately differentiated | 212 (30.6) | 167 (32.3) | 15 (17.1) | 30 (34.5) |  |
| Poorly differentiated/  Undifferentiated | 96 (13.9) | 71 (13.7) | 14 (15.9) | 11 (12.6) |  |
| Unknown | 384 (55.5) | 279 (54.0) | 59 (67.1) | 46 (52.9) |  |
| **Stage** |  |  |  |  |  |
| Regional | 459 (66.3) | 341 (65.0) | 60 (68.2) | 58 (66.7) |  |
| Distant | 233 (33.7) | 176 (34.0) | 28 (31.8) | 29 (33.3) |  |

Abbreviations: NOS, Not Otherwise Specified.
