# Supplementary material for: Treatment Outcomes Differ for Racial and Ethnic Minorities with Advanced-Stage Laryngeal Cancer: A Florida Cancer Data System Analysis
Source: Cancer Res Commun. 2025 Aug 11;5(8):1310–8. doi: 10.1158/2767-9764.CRC-25-0239 (PMC12336365; doi:10.1158/2767-9764.CRC-25-0239)
Supplement: Supplementary Table S3 — Cox proportional hazards models predicting risk of all-cause death for patients with advanced-stage laryngeal cancer who did not receive treatment, stratified by race and ethnicity and treatment strategy. [file crc-25-0239_supplementary_table_s3_suppst3.docx]

**Supplementary Table S3: Cox proportional hazards models predicting risk of all-cause death for patients with advanced-stage laryngeal cancer who did not receive treatment, stratified by race and ethnicity and treatment strategy.**

| **Race and Ethnicity** | **Crude Death Rate** | **Person-years** | **Death Rate**  **(Per 10 person-years)** | **Model 1** | **Model 2** | **Model 3** |
| --- | --- | --- | --- | --- | --- | --- |
| NH-White  NH-Black  Hispanic | 416/517  64/87  62/88 | 702  116  126 | 5.93 (5.4, 6.52)  5.52 (4.28, 7.00)  4.92 (3.81, 6.27) | REF  0.95 (0.73, 1.24)  0.77 (0.59, 1.01) | REF  0.88 (0.67, 1.15)  **0.71 (0.52, 0.95)** | REF  0.87 (0.67, 1.14)  **0.67 (0.50, 0.90)** |

Bolded values indicate P<0.05.

Model 1 adjusted for continuous age and sex.

Model 2 additionally adjusted for marital status, rurality, income, education, insurance,

Model 3 additionally adjusted for primary subsite and stage.

CI indicates confidence interval; HR, hazard ratio.

Crude death rate calculated by the number of deaths divided by the total number of individuals.

Death rate calculated as number of deaths divided by person-years (reported as cases/10-person years).
