# Supplementary material for: Treatment Outcomes Differ for Racial and Ethnic Minorities with Advanced-Stage Laryngeal Cancer: A Florida Cancer Data System Analysis
Source: Cancer Res Commun. 2025 Aug 11;5(8):1310–8. doi: 10.1158/2767-9764.CRC-25-0239 (PMC12336365; doi:10.1158/2767-9764.CRC-25-0239)
Supplement: Supplementary Table S4 — Sociodemographic and cancer characteristics for regional and distant staged laryngeal cancer patients who did not receive treatment. [file crc-25-0239_supplementary_table_s4_suppst4.docx]

**Supplementary Table S4: Sociodemographic and cancer characteristics for regional and distant staged laryngeal cancer patients who did not receive treatment.**

|  | **Overall (%)** |  | **Race n (%)** |  |
| --- | --- | --- | --- | --- |
|  | **N=3296** | **NH-White**  **N= 2966 (75.55)** | **Hispanic**  **N= 513 (13.1)** | **NH-Black**  **N= 447 (11.4)** |
| **Age** |  |  |  |  |
| <65 | 2258 (57.5) | 1688 (56.9) | 278 (54.2) | 292 (65.3) |
| 65+ | 1668 (42.5) | 1278 (43.1) | 235 (45.8) | 155 (34.7) |
| **Sex** |  |  |  |  |
| Male | 3121 (79.5) | 2307 (77.8) | 437 (85.2) | 377 (84.3) |
| Female | 805 (20.5) | 659 (22.2) | 76 (14.8) | 70 (15.7) |
| **Marital Status** | | | | |
| Married | 1688 (43.0) | 1345 (45.4) | 225 (43.9) | 118 (26.4) |
| Unmarried | 2238 (57.0) | 1621 (54.7) | 288 (56.1) | 329 (73.6) |
| **Primary insurance** | | | | |
| Private | 915 (23.2) | 713 (24.0) | 121 (23.6) | 81 (18.1) |
| Government | 2682 (68.3) | 2031 (68.5) | 333 (64.9) | 318 (71.1) |
| Not insured | 329 (8.4) | 222 (7.5) | 59 (11.5) | 48 (10.7) |
| **Rurality** |  |  |  |  |
| Metropolitan | 3707 (94.4) | 2784 (93.9) | 503 (98.1) | 420 (94.0) |
| Nonmetropolitan | 219 (5.6) | 182 (6.1) | 10 (2.0) | 27 (6.0) |
| **County Median Household Income** | | | | |
| <$48,000 | 2263 (57.6) | 1660 (56.0) | 357 (69.6) | 246 (55.0) |
| ≥48,000 | 1663 (42.4) | 1306 (44.0) | 156 (30.4) | 201 (45.0) |
| **Percentage of People in the County without High School Diploma** | | | | |
| 13% or more | 1256 (32.0) | 774 (26.1) | 344 (67.1) | 138 (30.9) |
| 7-12.9% | 2557 (65.1) | 2092 (70.5) | 162 (31.6) | 303 (67.8) |
| <7% | 113 (2.9) | 100 (3.4) | 7 (1.4) | 6 (1.3) |
| **Primary Subsite** |  |  |  |  |
| Glottis | 1044 (26.6) | 757 (25.5) | 178 (34.7) | 109 (24.4) |
| Supraglottis | 2106 (53.6) | 1636 (55.2) | 231 (45.0) | 239 (53.5) |
| Subglottis | 83 (2.1) | 67 (2.3) | 9 (1.8) | 7 (1.6) |
| Other/NOS | 693 (17.7) | 506 (17.1) | 95 (18.5) | 92 (20.6) |
| **Grade** |  |  |  |  |
| Well differentiated/Moderately differentiated | 1534 (39.1) | 1140 (38.4) | 209 (40.7) | 185 (41.4) |
| Poorly differentiated/  Undifferentiated | 724 (18.4) | 549 (18.5) | 98 (19.1) | 77 (17.2) |
| Unknown | 1668 (42.5) | 1277 (43.1) | 206 (40.2) | 185 (41.4) |
| **Stage** |  |  |  |  |
| Regional | 2559 (65.2) | 1980 (66.8) | 313 (61.0) | 266 (59.5) |
| Distant | 1367 (34.8) | 986 (33.2) | 200 (39.0) | 181 (40.5) |

Abbreviations: NOS, Not Otherwise Specified.

**Supplementary Table 5: Complete Case Analysis- Cox proportional hazards models predicting risk of all-cause death for patients with advanced-stage laryngeal cancer, stratified by race and ethnicity and treatment strategy.**

| **Race and Ethnicity** | | **Crude Death Rate** | **Person-years** | | **Death Rate**  **(Per 10 person-years)** | | **Model 1** | | **Model 2** | | **Model 3** | |
| --- | --- | --- | --- | --- | --- | --- | --- | --- | --- | --- | --- | --- |
| **Overall** | |  |  | |  | |  | |  | |  | |
| NH-White  NH-Black  Hispanic | | 2000/2966  321/447  301/513 | 7660  1029  1414 | | 2.61 (2.50, 2.73)  3.12 (2.79, 3.48)  2.13 (1.90, 2.38) | | REF  **1.22 (1.08, 1.37)**  **0.81 (0.71, 0.91)** | | REF  1.12 (1.00, 1.27)  **0.77 (0.68, 0.88)** | | REF  1.11 (0.98, 1.25)  **0.78 (0.69, 0.89)** | |
| **Chemoradiation** |  | | |  | |  | |  | |  | |  |
| NH-White  NH-Black  Hispanic | | 699/983  102/129  92/144 | 3257  378  487 | | 2.15 (1.99, 2.31)  2.70 (2.21, 3.26)  1.89 (1.53, 2.31) | | REF  **1.34 (1.09, 1.66)**  0.88 (0.71, 1.09) | | REF  1.24 (1.00, 1.53)  0.84 (0.67, 1.06) | | REF  **1.24 (1.01, 1.54)**  0.82 (0.65, 1.03) | |
| **Surgery and Chemoradiation** | | | | | | | | | | |  | |
| NH-White  NH-Black  Hispanic | | 169/235  34/41  29/52 | 855  116  196 | | 1.98 (1.70, 2.29)  2.93 (2.06, 4.05)  1.48 (1.01, 2.10) | | REF  1.37 (0.94, 1.99)  0.73 (0.49, 1.08) | | REF  1.38 (0.94, 2.03)  0.68 (0.45, 1.03) | | REF  1.31 (0.89, 1.92)  **0.62 (0.40, 0.95)** | |
| **Surgery and Radiation** | | | | | | | | | | |  | |
| NH-White  NH-Black  Hispanic | | 95/154  18/31  20/35 | 505  102  168 | | 1.88 (1.53, 2.29)  1.77 (1.08, 2.74)  1.19 (0.75, 1.81) | | REF  1.03 (0.62, 1.72)  **0.57 (0.35, 0.92)** | | REF  0.81 (0.46, 1.44)  0.60 (0.35, 1.02) | | REF  0.77 (0.43, 1.37)  0.58 (0.34, 1.00) | |
| **Radiation alone** | |  |  | |  | |  | |  | |  | |
| NH-White  NH-Black  Hispanic | | 160/190  23/28  16/24 | 480  50  69 | | 3.33 (2.85, 3.88)  4.60 (2.99, 6.79)  2.32 (1.37, 3,69) | | REF  1.34 (0.86, 2.08)  0.69 (0.41, 1.15) | | REF  1.21 (0.76, 1.92)  **0.53 (0.30, 0.94)** | | REF  1.05 (0.66, 1.69)  **0.51 (0.29, 0.91)** | |
| **Non-standard Treatment** | | | | | | | | | | |  | |
| NH-White  NH-Black  Hispanic | | 877/1404  144/218  144/258 | 2562  383  494 | | 3.42 (3.20, 3.66)  3.76 (3.18, 4.41)  5.58 (4.72, 6.55) | | REF  1.12 (0.94, 1.34)  0.84 (0.70, 1.00) | | REF  1.02 (0.86, 1.23)  0.83 (0.69, 1.00) | | REF   - 1. (0.84, 1.20)   0.86 (0.71, 1.04) | |

Bolded values indicate P<0.05.

Model 1 adjusted for continuous age and sex.

Model 2 additionally adjusted for marital status, rurality, income, education, insurance,

Model 3 additionally adjusted for primary subsite and stage. Overall model was adjusted for treatment.

CI indicates confidence interval; HR, hazard ratio.

Crude death rate calculated by the number of deaths divided by the total number of individuals.

Death rate calculated as number of deaths divided by person-years (reported as cases/10-person years).

Non-standard treatment includes chemotherapy alone, surgery alone, surgery followed by chemotherapy, and no treatment.
